# Prognostic Value of Magnesium in COVID-19: Findings from the COMEPA Study

**DOI:** 10.3390/nu15040830

**Published:** 2023-02-06

**Authors:** Anna La Carrubba, Nicola Veronese, Giovanna Di Bella, Claudia Cusumano, Agnese Di Prazza, Stefano Ciriminna, Antonina Ganci, Liliana Naro, Ligia J. Dominguez, Mario Barbagallo, on behalf of the COMEPA Group

**Affiliations:** 1 Department of Health Promotion, Mother and Child Care, Internal Medicine and Medical Specialties “G. D’Alessandro”, University of Palermo, 90127 Palermo, Italy; 2 School of Medicine and Surgery, University of Enna “Kore”, 94100 Enna, Italy

**Keywords:** magnesium, COVID-19, mortality, prognosis, long COVID, COMEPA

## Abstract

Magnesium (Mg) plays a key role in infections. However, its role in coronavirus disease 2019 (COVID-19) is still underexplored, particularly in long-term sequelae. The aim of the present study was to examine the prognostic value of serum Mg levels in older people affected by COVID-19. Patients were divided into those with serum Mg levels ≤1.96 vs. >1.96 mg/dL, according to the Youden index. A total of 260 participants (mean age 65 years, 53.8% males) had valid Mg measurements. Serum Mg had a good accuracy in predicting in-hospital mortality (area under the curve = 0.83; 95% CI: 0.74–0.91). Low serum Mg at admission significantly predicted in-hospital death (HR = 1.29; 95% CI: 1.03–2.68) after adjusting for several confounders. A value of Mg ≤ 1.96 mg/dL was associated with a longer mean length of stay compared to those with a serum Mg > 1.96 (15.2 vs. 12.7 days). Low serum Mg was associated with a higher incidence of long COVID symptomatology (OR = 2.14; 95% CI: 1.30–4.31), particularly post-traumatic stress disorder (OR = 2.00; 95% CI: 1.24–16.40). In conclusion, low serum Mg levels were significant predictors of mortality, length of stay, and onset of long COVID symptoms, indicating that measuring serum Mg in COVID-19 may be helpful in the prediction of complications related to the disease.

## 1. Introduction

Magnesium (Mg) is the most frequent divalent cation present intracellularly in the human body and the second most common intracellular ion after potassium [1]. Mg is essential for numerous cellular processes because it is a cofactor of over 600 intracellular enzymatic reactions [2]. Magnesium is essential for energy production, oxidative phosphorylation, glycolysis, protein synthesis, and nucleic acid synthesis and stability [3,4]. Magnesium modulates muscle contraction, normal heart rhythm and neuron excitability, as it is necessary for the transport of other ions across cellular membranes [5]. This essential ion is involved in all ATP-dependent biochemical processes as part of the activated MgATP complex, as well as in RNA expression, DNA synthesis, muscular and neural cellular signaling, glucose metabolism and blood pressure control [6,7].

Low serum Mg concentrations, rather common in the Western world, are frequently observed in older people, because of poor intake in the diet, some comorbidities (e.g., diabetes), and polypharmacy [8,9,10,11]. Older adults, together with a higher frequency of Mg deficiency, suffer alterations to the immune system that make them more susceptible to infections and their complications [12]. This includes the increased risk of major complications and especially mortality, which has been observed in SARS-CoV-2 infection [13].

Low Mg levels play an important role in several chronic diseases affecting older people, including respiratory conditions, such as chronic obstructive pulmonary disease (COPD) and asthma [14,15]. A former retrospective study reported that serum Mg was an independent predictor of frequent readmissions for acute COPD exacerbations [16]. More recently, a study showed that low Mg status was predictive of the risk of bacterial pneumonia in old age [17]. Furthermore, other studies indicated that low serum Mg levels were present in asthmatic patients [18], and that Mg supplementation may be useful in the treatment of acute asthma [19]. This is based on the evidence that bronchial hyperreactivity is inversely proportional to the serum level of this cation [20,21].

Mg plays a role in the immune system, in both innate and acquired immune response, being involved as a cofactor for immunoglobulin (Ig) synthesis, C3 convertase, immune cell adherence, antibody-dependent cytolysis, IgM lymphocyte binding, macrophage response to lymphokines, and T helper–B cell adherence [22]. A recent study showed that altered Mg status seems to have a prognostic role in older people affected by bacterial pneumonia [17]. Of interest, both hypomagnesemia and hypermagnesemia were associated with an increased short-term mortality rate compared to normal values of serum Mg in these patients with community acquired pneumonia [17]. Vitamin D, which seems to play a key role in the immune response [23], requires an adequate level of Mg for its proper transport and activation [5,24,25]. Thus, a Mg deficiency can exacerbate susceptibility to infections, including COVID-19, by reducing the availability or adequate functional levels of vitamin D.

Recently, some works explored the possible role of Mg in COVID-19 to predict the prognosis in these patients [26,27,28,29,30]. However, no study so far has examined the role of Mg for long COVID prediction, an increasing entity with limited therapeutical options [31]. The present study, exploring the prognostic value of serum Mg in patients with COVID-19, follows one of the original aims of the COMEPA study, which was to explore possible prognostic factors for COVID-19 complications during or after hospitalization based on our real-life experience [32]. Validated markers capable of predicting the variable trajectory of the disease from completely asymptomatic to mild, moderate or several clinical manifestation and rapidly progressive forms that can lead to multiorgan failure and death have not yet fully identified. In fact, although several proposed prognostic models have been reported, most of them are of poor quality [33], highlighting the need to continue research on useful tools with this specific aim.

In consideration of the role of Mg in the most frequent respiratory diseases, including infections, and due to the lack of literature in this regard, the purpose of our study was to examine the prognostic value of serum Mg in patients affected by COVID-19, in terms of in-hospital mortality, length of stay, and the occurrence of long COVID.

## 2. Materials and Methods

### 2.1. Study Population

All patients aged ≥18 years hospitalized in the internal medicine or geriatrics wards from the 1st of September 2020 at the University Hospital ‘*p*. Giaccone’ in Palermo, Italy with a diagnosis of SARS-CoV-2 infection confirmed by the observation of SARS-CoV-2 nucleic acid on a nasopharyngeal swab by means of RT-PCR were enrolled [32]. No other inclusion criteria were considered to better represent a real-life scenario. The study was approved by the Local Ethical Committee during the session of the 28th of April 2021 (protocol number 04/2021). For hygiene reasons, the informed consent to participate in the study was collected orally and reported in the medical records.

### 2.2. Exposure: Serum Mg Levels

Serum Mg levels at the baseline were measured in the first four days of the hospital admission, including measurements at the emergency department. The values of serum Mg assessed during hospitalization were also recorded and the maximum value during the hospital stay was used as covariate for the analyses. The normal range of our laboratory for serum Mg was 1.7 to 2.5 mg/dL.

### 2.3. Outcomes

The primary outcome was mortality during hospital stay. This information was collected using dates of death according to the clinical records and death certificates. As secondary outcomes, we considered the length of stay in hospital and the incidence of long COVID symptomatology. In October 2021, the World Health Organization (WHO) defined long COVID as “*a condition that occurs in individuals with a history of probable or confirmed SARS-CoV-2 infection, usually 3 months from the onset of COVID-19 with symptoms that last for at least 2 months and cannot be explained by an alternative diagnosis*”. [34] Accordingly, the presence of long COVID was assessed after a median of 17 months (range: 13–22) from hospital discharge through phone calls similar to other works using the same approach [35,36,37,38]. We considered as signs or symptoms of long COVID those indicated in recent systematic reviews [35,36,37,38], i.e., neurological, respiratory, mobility impairment, heart, digestive, skin, or general signs and symptoms that can be attributable to COVID-19 infection. All of the questions were posed as yes/no questions by phone. Psychiatric conditions were assessed using the Post-traumatic Stress Disorder (PTSD) Checklist (PCL)-5 [39] and the Hospital Anxiety and Depression Scale (HADS) for detecting anxiety and depression [40].

### 2.4. Covariates

Among the parameters that were collected in the COVID-19 Palermo (COMEPA) study [32], for the aim of the present study, we used the information potentially affecting the association between serum Mg levels and the outcomes of interest, i.e., age, gender, smoking status (actual vs. previous or never), and alcohol abuse (yes vs. no). Among laboratory measurements, we considered creatinine clearance according to the Modification of Diet in Renal Disease (MDRD) formula, hemoglobin, serum parameters of inflammation (white blood cells, C reactive protein (CRP), interleukin (IL)-6, procalcitonin), parameters of arterial blood gas exchange expressed as partial pressure of oxygen/fraction of inspired oxygen (PaO_2_/FiO_2_) ratio (with a value below 150 indicative of acute respiratory failure) [41], serum 25 hydroxyvitamin D (25OHD), hepatic function, fasting plasma glucose, sodium, potassium, and albumin. The presence and the severity of comorbidities were investigated using the Cumulative Illness Rating Scale (CIRS) [42] that estimates the severity of pathology in each of 13 systems, with a grade from 0 to 4 (severity index: CIRS-SI).

### 2.5. Statistical Analyses

All patient records and information were anonymized and de-identified prior to the analyses. We selected the cut-off value of 1.96 mg/dL of serum Mg since it was the best in terms of sensitivity and specificity (Youden’s index) [43] for testing the prediction of our primary outcome. Data on continuous variables were normally distributed according to the Kolmogorov–Smirnov test and then reported as means and standard deviation (SD) values for quantitative measures and percentages for the categorical variables, by serum Mg status. Levene’s test was used to test the homoscedasticity of variances and, if its assumption was violated, Welch’s ANOVA was used. *p* values were calculated using Student’s *t*-test for continuous variables and the Mantel–Haenszel chi-square test for categorical ones.

The accuracy of serum Mg in predicting in-hospital mortality during follow-up was calculated in terms of area under the curve (AUC) with its 95% confidence intervals (CIs). The association between serum Mg at baseline being less or more than 1.96 mg/dL and in-hospital mortality was assessed using Cox’s regression analysis, adjusted for potential confounders that were introduced in the model if they did differ between low and high serum Mg levels (*p*-value < 0.05) or if they were associated with in-hospital death using a *p*-value threshold of 0.10. Collinearity among factors was analyzed using a variance inflation factor (VIF) of two as a reason for exclusion. The results, considering participants with serum Mg over 1.96 mg/dL as a reference, were reported as hazard ratios (HRs) with their 95% confidence interval (CI). Data regarding long COVID were reported using an adjusted logistic regression and reported as odds ratios (ORs) with their 95% CI.

All analyses were performed using the SPSS 26.0 for Windows (SPSS Inc., Chicago, IL, USA) and STATA 14.0. All statistical tests were two-tailed and statistical significance was assumed for a *p*-value < 0.05.

## 3. Results

Among 530 patients initially included in the COMEPA study, 270 were excluded: 250 had not any serum Mg measurement in the first four days from admission and the other 20 did not register any of the outcomes of interest. Consequently, a total of 260 participants (mean age 65.4 ± 15.4, range: 21–96 years; 53.8% men) were included in the analyses. The mean serum Mg level was 2.07 ± 0.23 mg/dL (range: 1.32–2.50), with 26 patients (10.0%) reporting hypomagnesemia identified as a value <1.85 mg/dL. In cases of hypomagnesemia, the patients were supplemented using intravenous Mg sulfate, with the dose depending on the severity of hypomagnesemia until the normalization of Mg serum concentrations. No one reported hypermagnesemia (serum Mg > 2.5 mg/dL).

Table 1 shows the baseline characteristics according to the serum Mg levels. The 74 patients with a value less than 1.96 mg/dl were significantly older (*p* = 0.01), but they did not differ in terms of the percentage of males or in their smoking prevalence or alcohol abuse, compared to their counterparts with higher serum Mg levels. Among the laboratory parameters assessed, patients with low serum Mg levels displayed significantly lower hemoglobin and albumin levels, but they did not differ in any of the inflammatory parameters investigated (Table 1). Finally, patients with low serum Mg levels reported a significantly lower prevalence of any COVID-19 symptomatology, but a higher severity of medical conditions, according to the CIRS-SI.

**Figure 1:**
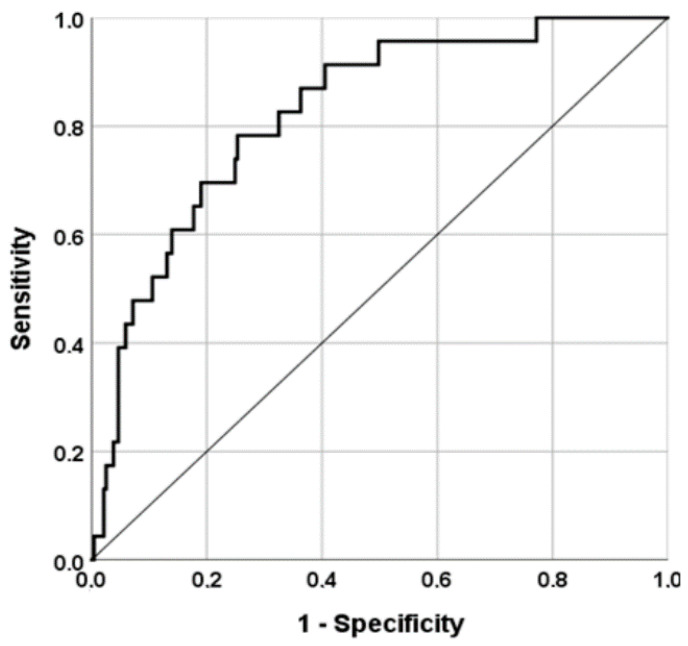
Accuracy of serum Mg at admission in predicting in-hospital mortality.

As shown in Figure 1, a model including serum Mg, adjusted for age and sex, had a good accuracy in predicting in-hospital mortality (AUC = 0.83; 95% CI: 0.74–0.91; *p* < 0.0001). A value of serum Mg = 1.96 during the first four days of hospitalization had a good sensitivity (75%) and a modest specificity (58%) in predicting mortality during hospital stay.

**Figure 2:**
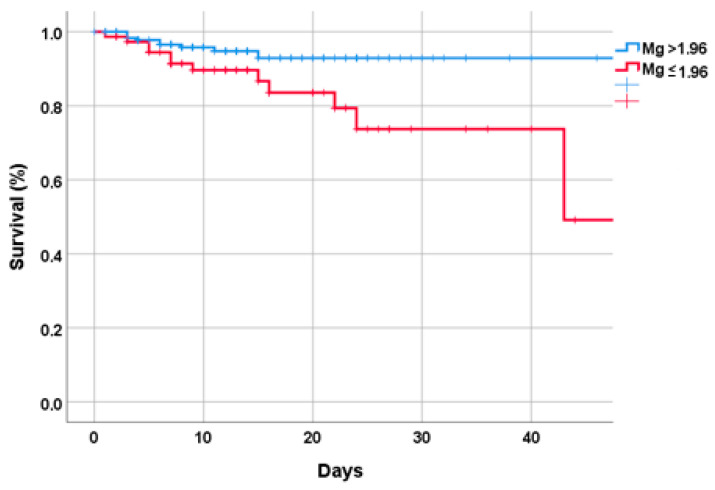
Survival status by serum Mg status at the baseline.

Low serum Mg at admission significantly predicted in-hospital death (HR = 1.29; 95% CI: 1.03–2.68) after adjusting for age, sex, comorbidities, renal function, presence of respiratory failure, CRP, hemoglobin, and maximum Mg serum levels during hospitalization (Figure 2). A value of Mg ≤ 1.96 was associated with a longer mean length of stay compared to those with a serum Mg > 1.96 (15.2 vs. 12.7 days; *p* = 0.048).

Finally, we investigated the association between low serum Mg levels and the presence of long COVID among 95 patients with available data. Among all the signs and symptoms investigated, low serum Mg was associated with a higher incidence of overall long COVID symptomatology (OR = 2.14; 95% CI: 1.30–4.31), particularly PTSD (OR = 2.00; 95% CI: 1.24–16.40), whilst no significant association was found for the other single long COVID signs/symptoms investigated in our questionnaire.

## 4. Discussion

Our study including 260 participants hospitalized for COVID-19 indicates the important role of Mg in the prognosis of these patients. Patients with lower serum Mg levels, in fact, had an increased risk not only of in-hospital mortality, but also a longer length of stay and higher incidence of long COVID symptomatology, with this study being, to the best of our knowledge, the first to confirm these significant associations.

In our study, at hospital admission the prevalence of patients with hypomagnesemia was high, i.e., 10%. Patients with lower serum Mg levels reported some baseline characteristics that could increase the risk of mortality, including older age, significantly lower hemoglobin and albumin levels, and a higher comorbidity and severity of medical conditions. However, after adjusting for all these parameters, the association between lower serum Mg and in-hospital mortality remained statistically significant, indicating an independent role of Mg in poor prognosis among patients hospitalized for COVID-19. Previous studies have reported the prognostic importance of low serum Mg in COVID-19. Of interest, in a large North American population, it was reported that the infection risk for COVID-19 of the populations distributed in low-Mg areas was higher than those introducing a higher intake of dietary Mg [44]. Moreover, other studies reported an important role of Mg in the prognosis of patients affected by COVID-19. For example, Guerrero-Romero et al. analyzed 1064 patients with COVID-19, showing a significant association between serum magnesium-calcium ratio and mortality in severe forms of the disease [27]. Similarly, a retrospective cohort study analyzing 390 hospitalized patients with COVID-19 showed that reduced kidney function and lower serum Mg levels were associated with increased mortality in obese patients affected by COVID-19 [30]. In addition, Zeng et al. performed a retrospective study, analyzing 306 patients with COVID-19 for their whole blood levels of essential minerals, including Mg, and found that severe cases showed significantly lower levels of Mg than mild and moderate cases [29]. All these reported findings, associated with the results of our study, indicates that Mg might play a key role in maintaining proper immune, vascular and lung function. This strongly supports the hypothesis on which several studies have been based, that serum Mg status may influence susceptibility and response to SARS-CoV-2 infection [5].

As mentioned, our study confirmed the significant prognostic value in COVID-19 patients already reported in previous studies [26,27,28,29,30]. There are several mechanisms that may help to explain the link between a low Mg status and an increased risk of severe forms of the disease and mortality. COVID-19 is now considered a potential systematic disease due to the possibility not only of leading to acute respiratory distress syndrome requiring hyperoxic ventilation, but also of impacting other organs and systems, comprising the cardiovascular, hepatic, intestinal, renal and nervous systems [45]. Older adults are more susceptible to severe illness, ICU admission, and mortality from COVID-19 [46]. This trend has been confirmed since the beginning of the pandemic and it is particularly high in older adults with multimorbidity. Although the ultimate mechanisms of COVID-19 clinical manifestations and mortality are not completely clear, the cytokine storm seems to contribute significantly to the pathogenesis of the most severe manifestations of the disease [45]. Cytokine storm refers to the overproduction of soluble markers of inflammation that maintain an aberrant response of systemic inflammation. It seems that the collateral damage caused by the excessive production of inflammatory mediators, in an attempt to eliminate the pathogen, may be more damaging than the pathogen itself. Indeed, this exuberant inflammatory response may initially be appropriate to control the infection, but if uncontrolled and persistent, it can fuel the multi-organ dysfunction that may follow, increasing the risk of mortality. The cascade of inflammatory mediators during cytokine storm includes immunoactive molecules, e.g., interferon, chemokines, interleukins, TNF-alpha, and colony-stimulating factors [47].

There is extensive evidence in experimental investigations [48,49,50,51] as well as in observational studies in humans [52,53,54,55,56,57] confirming that a low Mg status is associated with a state of chronic inflammation with increased inflammatory markers, particularly IL-6, TNF-alpha, and the complex IL-33/ST2. Furthermore, some studies have reported anti-inflammatory actions of Mg supplementation and suppression of cytokine release [58,59]. A meta-analysis including eight RCTs reported a significant reduction in serum CRP after Mg supplementation, which was independent of Mg dosage or the length of follow-up [60]. A well-known action of Mg is its antagonistic effects on calcium channels [61,62]. Indeed, Mg is considered a natural calcium blocker, similar to those of chemical synthesis [63]. Interestingly, calcium channel blocking effects of Mg can lead to the suppression of NF-kB, IL-6, and CRP [59], which may limit systemic inflammation.

Patients with severe forms of COVID-19 may need ICU admission. Remarkably, up to 60% of critically ill patients in the ICU have some degree of Mg deficiency [64,65], which makes them more susceptible to potentially fatal effects, also associated with the consequent hypokalemia and hypocalcemia. Perhaps the lack of attention to paid Mg in COVID-19 may be due to the fact that it is not routinely measured in most databases and studies [66]. In addition, serum concentrations that are clinically available represent only 1% of the total body Mg and do not accurately reflect the whole Mg status, being this ion predominantly intracellular [9].

Thus, the preceding Mg deficiency associated with conditions that favor a detrimental course of COVID-19, including age, diabetes, hypertension [6,9,15] and the Mg deficiency frequently observed in critically ill patients [64,65], can contribute to exacerbate the inflammatory response induced by SARS-CoV-2, which in turn can determine an increased Mg consumption, resulting in a further reduction in its intracellular levels, maintaining and propagating an uncontrolled inflammatory response.

The evidence that COVID-19 pneumonia and multi-organ dysfunction has a vascular basis is robust [67,68]. The vascular endothelium is crucial in the maintenance of homeostasis and the control of fibrinolysis, inflammation, vasomotion, oxidative stress, vascular permeability and structure. All of these functions acting in concert regulate many of the defense mechanisms against external noxae, but they can also contribute to disease at different levels when the usual homeostatic functions are overwhelmed and turn against the host, as has been reported in COVID-19 [69]. There is also convincing evidence that Mg has antithrombotic effects [70], while low Mg concentrations have been associated with endothelial dysfunction [71,72]. A systematic review and meta-analysis of RCTs exploring the effects of Mg supplementation on vascular function showed that oral Mg supplementation significantly improved flow-mediated dilation in studies lasting longer than 6 months, including healthy people, older than 50 years, or with BMI greater than 25 kg/m^2^ [73]. Hence, it is possible that a chronic Mg deficiency, common in older adults [9], may generate a favorable environment for SARS-CoV-2 to promote thrombosis [66], a fundamental characteristic of COVID-19.

It is widely known that Mg plays a role in the immune system, in both innate and acquired immune response [22], and this effect is probably of importance in COVID-19, often characterized by a decreased immune response. In fact, Mg is a cofactor for the synthesis of immunoglobulins (Ig), as well as for C3 convertase, antibody-dependent cytolysis, immune cell adherence, macrophage response to lymphokines, IgM lymphocyte binding, and T helper–B cell adherence [22,74]. Mg induces the reduction in proinflammatory molecule release, such as P, by controlling nuclear factor kappa-light-chain-enhancer of activated B cell NF-kB activity [75]. In addition, Mg affects acquired immunity by regulating lymphocyte development and proliferation [76]. There is evidence that experimental animals fed Mg-deficient diets showed altered polymorphonuclear cell number and function, as well as increased phagocytosis [77]. Mast cell proliferation and function are also modified by Mg deficiency [78]. In addition, Mg deficit has been involved in mast cell-dependent hepatic fibrosis and steatosis [79]. In addition, Fas-induced B cell apoptosis is a Mg-dependent process [80]. Other studies have confirmed that Mg-deficient experimental animals exhibited high rates of inflammation and reduced specific immune responses [51,81,82,83]. The increased inflammation associated with Mg deficiency in old age [9] has been linked to several mechanisms, including opening of calcium channels, activation of phagocytic cells, activation of N-methyl-d-aspartate (NMDA) receptor and of NF-kB [48]. The best evidence of the fundamental role of Mg as a second messenger in immunity was the discovery of a genetic disease, X-linked immunodeficiency with magnesium defect (XMEN), which can lead to severe and chronic Epstein–Barr virus infections and neoplasia [84,85,86].

Moreover, due to its vasodilatory, anti-inflammatory and anti-thrombotic effects, the role of Mg was recently explored in COVID-19 patients [26]. All these effects, in fact, might contribute to the reduction in the ventilation-perfusion mismatch, which is one of the most important reasons for hypoxemia in COVID-19, and to the improvement of oxygenation in these patients [87]. Additionally, because of the emerging role of mastocytes in driving diffuse alveolar injury in COVID-19 [88], it should be recalled that Mg may reduce mastocyte degranulation and, subsequently, prevent the release of inflammatory, pro-thrombotic and fibrotic mediators [89].

We believe that our study adds novel information to the current literature regarding Mg in COVID-19 debate. Low serum Mg levels not only were associated with a higher mortality risk during hospitalization and improved the accuracy of the prediction of this outcome among hospitalized patients, but also predicted a longer length of stay in hospital and a higher incidence of long COVID. To the best of our knowledge, our study is the first to show the impact of low serum Mg status for long COVID and, in particular, for PTSD. Since long COVID may affect more than 50% of the patients previously hospitalized for COVID-19 [90], our study suggests the need to early identify and correct poor Mg status in order to help prevent this complication. Of importance, our study suggests that a peculiar association with psychiatric disorders may exist, confirming the previous literature in this direction [91].

The findings of our study must be interpreted within its limitations. First, a consistent part of the initially considered population was not included, since data regarding serum Mg were not always available. Therefore, a selection bias cannot be ruled out. Second, long COVID was detected using phone calls and not using other more validated tests, such as medical records. We have recently had the opportunity to review systematically and perform a meta-analysis of the incidence and frequency of signs and symptoms of long COVID according to the definition of the World Health Organization among 120,979 patients from 196 studies, as shown below [90]. In the [Supplementary Table S2 of the article, we report the characteristics of the 196 studies included, comprising the methods of follow-up assessing the symptomatology for the formulation of long COVID diagnosis. Among the 196 studies, 51 (26%) used phone calls, 90 (45.9%) used an outpatient visit, 18 (9.2%) used an online electronic survey, 13 (6.6%) used an in-person interview, 15 (7.7%) used a mixed method, 5 (2.6%) used other methods, and 4 (2%) did not specify any method. Therefore, about one-quarter of published studies used phone calls, the method we used in our study. The use of these methods, which in almost half of the cases did not involve a classic outpatient visit, is understandable due to the conditions of the pandemic and the measures for containing its spread in accordance with WHO and with all the health systems worldwide in unique conditions. This was the way to be able to continue with the investigations. Third, even if we clearly asked if a sign or symptom could be independent of COVID-19 during the follow-up, we cannot exclude the possibility that the symptomatology could be attributed to other concurrent issues. It must also be considered that in clinical practice, only serum Mg assessment is available, which may not accurately reflect the total body Mg status, with Mg being a prevalently intracellular ion. Finally, even if highly prevalent in percentage (10%), only 26 patients had hypomagnesemia at the baseline, making the research of potential risk factors associated with this condition very difficult.

## 5. Conclusions

Our study indicates the importance of low serum Mg levels in the prognosis of COVID-19 complications, not only for predicting mortality and a longer length of stay in hospital, but also for the prediction of a higher presence of long COVID, even if this latter condition was ascertained using phone calls. Therefore, we warmly recommend that serum Mg be determined in all patients admitted for COVID-19. Further studies involving Mg supplementation are needed to determine if this intervention can indeed alter the course of the disease in a selected cohort.

## Figures and Tables

**Table 1:** Descriptive baseline characteristics by Mg status at admission: the COMEPA study.

Parameter	Serum Mg > 1.96 mg/dL (*n* = 186)	Serum Mg ≤ 1.96 mg/dL (*n* = 74)	*p*-Value
***Demographics***			
**Age (years)**	63.9 (15.7)	69.1 (13.4)	0.01
**Males (%)**	51.6	59.5	0.27
**Current smoking (%)**	12.1	9.8	0.80
**Alcohol abuse (%)**	6.1	10.4	0.34
***Laboratory parameters (1–4 days)***			
**Creatinine clearance (mL/min)**	91.7 (32.0)	87.8 (45.5)	0.50
**Hemoglobin (g/dL)**	13.1 (1.9)	11.6 (2.2)	<0.0001
**White blood cells (units/μL)**	7940 (4214)	7212 (3264)	0.14
**CRP (mg/mL)**	51.2 (48.9)	60.0 (64.6)	0.31
**IL-6 (pg/mL)**	36.4 (71.0)	59.8 (136.1)	0.19
**Procalcitonin (ng/mL)**	0.82 (6.84)	0.43 (1.26)	0.68
**PaO_2_/FiO_2_ ratio**	324 (104)	344 (142)	0.29
**Serum 25OHD (ng/mL)**	24 (19)	23 (14)	0.78
**AST (U/L)**	31 (25)	26 (15)	0.21
**ALT (U/L)**	37 (46)	25 (19)	0.003
**Fasting plasma glucose (mg/dL)**	131 (62)	141 (71)	0.29
**Na (mmol/L)**	139 (4)	139 (4)	0.88
**Ca++ (mmol/L)**	8.94 (0.93)	8.95 (0.63)	0.99
**K (mg/dL)**	4.38 (0.67)	4.43 (0.58)	0.10
**Albumin (g/L)**	3.57 (0.51)	3.36 (0.47)	0.002
***Clinical data***			
**Any COVID-19 symptomatology (%)**	82.3	63.0	0.002
**CIRS-SI**	1.41 (1.42)	2.74 (1.81)	<0.0001

AST: aspartate aminotransferase; ALT; alanine transaminase; Ca: calcium; CIRS-SI: Cumulative Illness Rating Scale-Severity Index; COMEPA: COVID-19 Palermo; COVID-19: coronavirus-19 disease; CRP: C-reactive protein; IL-6: interleukin-6; K: potassium; Na: sodium; PaO_2_/FiO_2_: partial pressure of oxygen/fraction of inspired oxygen; 25OHD: 25 hydroxyvitamin D.

## Data Availability

The data are available upon reasonable request to the corresponding author.
